# Supplementary material for: Ectopic Expression of HbRPW8-a from Hevea brasiliensis Improves Arabidopsis thaliana Resistance to Powdery Mildew Fungi (Erysiphe cichoracearum UCSC1)
Source: Int J Mol Sci. 2022 Oct 20;23(20):12588. doi: 10.3390/ijms232012588 (PMC9603905; doi:10.3390/ijms232012588)
Supplement: Supplementary file 1 [file ijms-23-12588-s001.zip › ijms-1925878-supplementary.pdf]

Supplemental Table S1: Gene and protein information

Genes	Assesion	ORF lenth (bp)	Number of exon	Number of intron	Number of AA	Molecular weight(D)	Theoretical pI hydropathicity
HbRPW8-a	XM_021812317.1	570	1	0	189	21728.51	9.23
HbRPW8-b	XM_021802913.1	2427	5	4	808	90347.87	5.77
HbRPW8-c	XM_021802915.1	2454	5	4	817	91902.35	5.92
HbRPW8-d	XM_021812335.1	2457	1	0	818	92820.43	6.41

Supplemental Table S2: Primers used in this study

primer name	Primers sequence (5'→3')	Description
*HbRPW8-a*qRT-F	GAAGATCTTCAAGCTAGATTTGTTTCATAGC	qRT-PCR
*HbRPW8-a*qRT-R	GGACTAGTCCCCAAAAGCGAATTTCTAAGCA	
*HbRPW8-b*qRT-F	CCAAGGAGTGTTACATGGACCTA	qRT-PCR
*HbRPW8-b*qRT-R	TACCAATAGCCTCTTTCTGTGCT	
*HbRPW8-c*qRT-F	CCAAGGAGTGTTACATGGACCTA	qRT-PCR
*HbRPW8-c*qRT-R	CCTGAGCTGCTTTGAATGATAG	
*HbRPW8-d*qRT-F	ATGTGGGCAGAACTGTATGAACT	qRT-PCR
*HbRPW8-d*qRT-R	AAGAAATCGGGTAAACTGTAGGT	
Hb18s-F	CCTTACTACTTGGATAACCGTGG	qRT-PCR (internal
Hb18s-R	CATCGAAAGTTGATAGGGCAG	reference gene)
AtRPW8.2-F	ATGATTGCTGAGGTTGCCGCAG	For AtRPW8.2 transient
AtRPW8.2-R	AGAATCATCACTGCAGAACGTAAATCG	expression
AtActin2-F	AGTGTCTGGATCGGTGGTTC	qRT-PCR (internal
AtActin2-R	AAAGGCTTAAAAAGCTGGGG	reference gene)
HbRPW8-a-F	ATGGAAATGGCTGAAGAGGC	For *HbRPW8-a*
HbRPW8-a-R	CCAAAAGCGAATTTCTAAGCATT	overexpression

Supplemental Table S3: The accession numbers of various RPW8 gene

Species	Gene	Assesion/ Gene number	pfam domain (HMMER)	Reference
Vitis pseudoreticulata	*VpRPW8-a*	KU365990	RPW8-NR-ARC-LRR	[41]
	VpRPW8-b	KU365991	RPW8-NR-ARC-LRR	
	VpRPW8-c	KU365992	RPW8-NR-ARC-LRR	
	VpRPW8-d	KX389173	RPW8-NR-ARC,	
	VpRPW8-e	KX389175	RPW8-NR-ARC-LRR	
Brassica_napus	*HRa*	KF906142	RPW8	[40]
	HRb	KJ476911		
	HRc	KJ476927		
	HRd	KJ476940		
strawberry (Fragaria ×ananassa Duchase)	*RPW8*	mrna24125.1-v1.0-hybrid	RPW8-NB-ARC	[42]
		mrna28370.1-v1.0-hybrid	RPW8-(NB-ARC)2	
		mrna08419.1-v1.0-hybrid	NB-ARC-RPW8	
		mrna28348.1-v1.0-hybrid	(NB-ARC)2 CC RPW8	
		mrna24108.1-v1.0-hybrid	RPW8 NB-ARC, CC, Pkinase_Tyr- Pkinase	
		mrna19978.1-v1.0-hybrid	NB-ARC, RPW8, zf-RING_2 (Ring finger domain)	
		mrna24089.1-v1.0-hybrid	RPW8-CC NB-ARC- Peptidase_C48	
Cucurbita pepo L.	*Cp4.1LG10g02780*	NOT found	RPW8- GRAM	[41]
	Cp4.1LG10g02800		RPW8	
	Cp4.1LG10g02750		RPW8-NR-ARC-LRR	
Akebia trifoliata	*AtNBS51*	NOT found	RPW8-NR-ARC-LRR	[62]
	AtNBS49			
	AtNBS50			
	AtNBS52			
Lagenaria siceraria	*Lsi01G010000.1*	NOT found	RPW8-NR-ARC-LRR	[63]
	Lsi11G004730.1			
	Lsi04G015960.1			

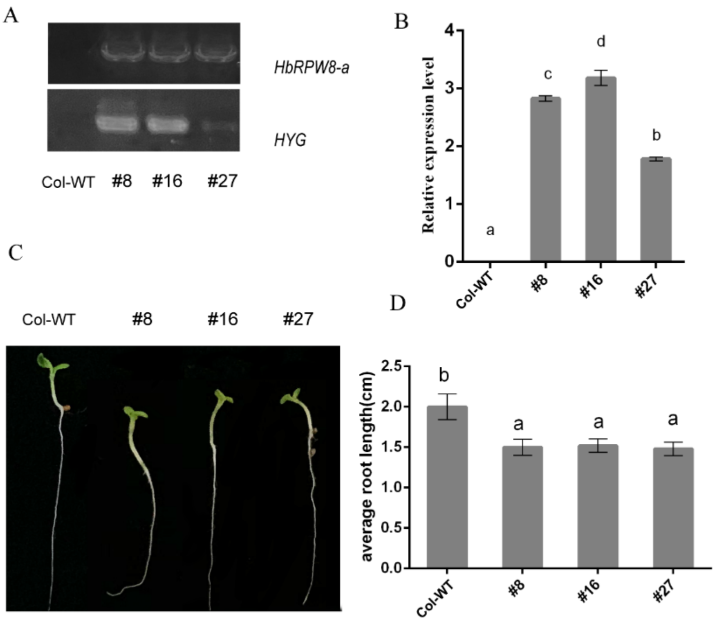

Supplemental Figure S1. Phenotypes of *Arabidopsis* transgenic lines with heterologous overexpression of *HbRPW8-a*

A Expressional analysis of the *HbRPW8-a* and positive control *Hygromycin* genes (*HYG*), which was assembled in the plant expression vector used in this experiment to detect the presence of the expression vector in the plant, by reverse transcription polymerase chain reaction in the Col-WT, and transgenic lines. B Real-time quantitative PCR analysis of the relative expression levels of *HbRPW8-a* gene in *Arabidopsis* overexpression lines (#8, #16, #27) and Col-WT. C Representative image of *Arabidopsis* after initial growth in 1/2MS medium. D Average root length of Col-WT and transgenic lines (#8, #16, #27). Data represent the mean ± standard deviation (SD) of three independent experiments. * indicates the difference between the transgenic line and the Col-WT. (P<0.05, t-test).

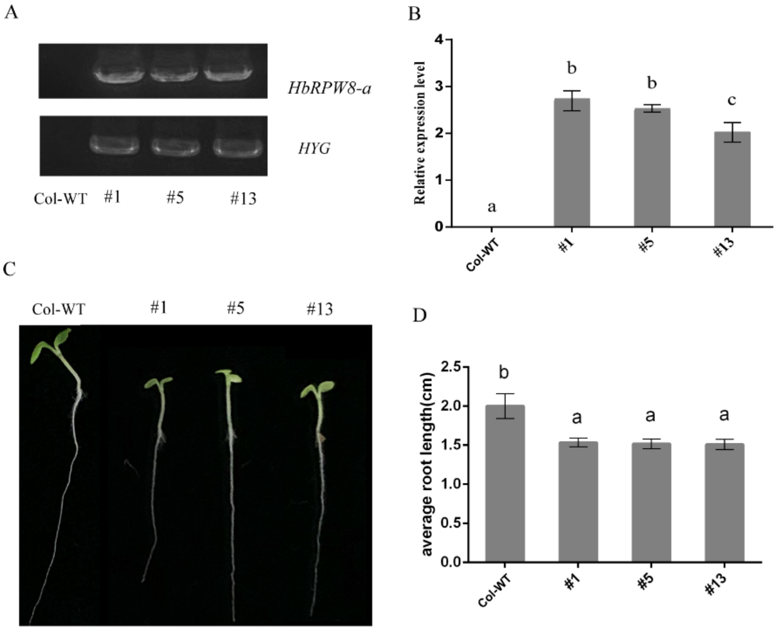

Supplemental Figure S2. Phenotypes of *HbRPW8-a* overexpressed lines at *NahG* background

A Expressional analysis of the *HbRPW8-a* and positive control Hygromycin genes (HYG), which was assembled in the plant expression vector used in this experiment to detect the presence of the expression vector in the plant, by reverse transcription polymerase chain reaction in the Col-WT, and transgenic lines. B Real-time quantitative PCR analysis of the relative expression levels of *HbRPW8-a* gene

in *Arabidopsis* overexpressing lines (#1, #5, #13) and Col-WT. C Representative image of *Arabidopsis* after initial growth in 1/2MS medium. D Average root length of Col-WT and transgenic lines (#1, #5, #13). Data represent the mean ± standard deviation (SD) of three independent experiments. * indicates the difference between the transgenic line and the Col-WT (P<0.05, t-test).

The raw gel image of SF1

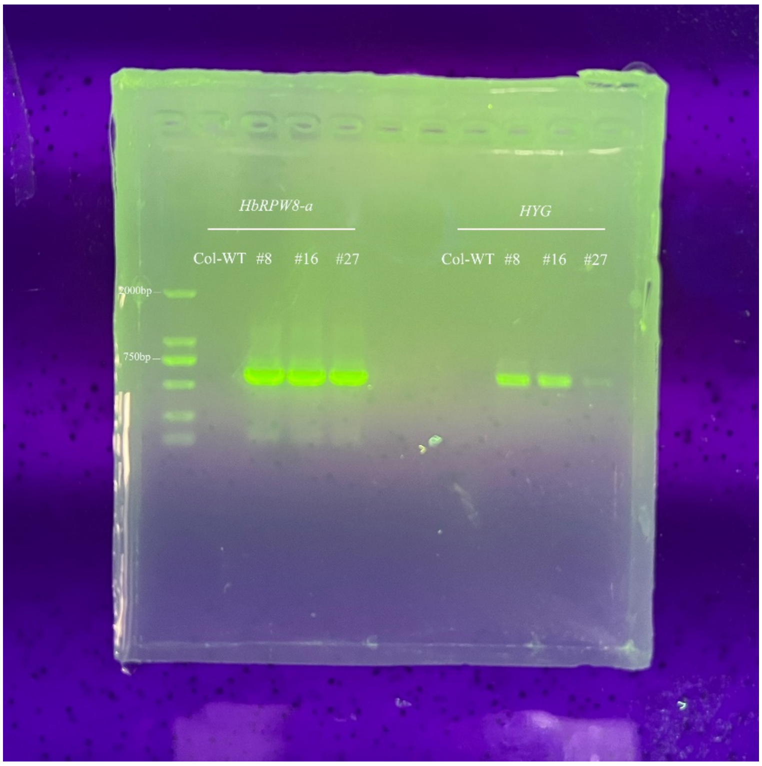

The raw gel image of SF2

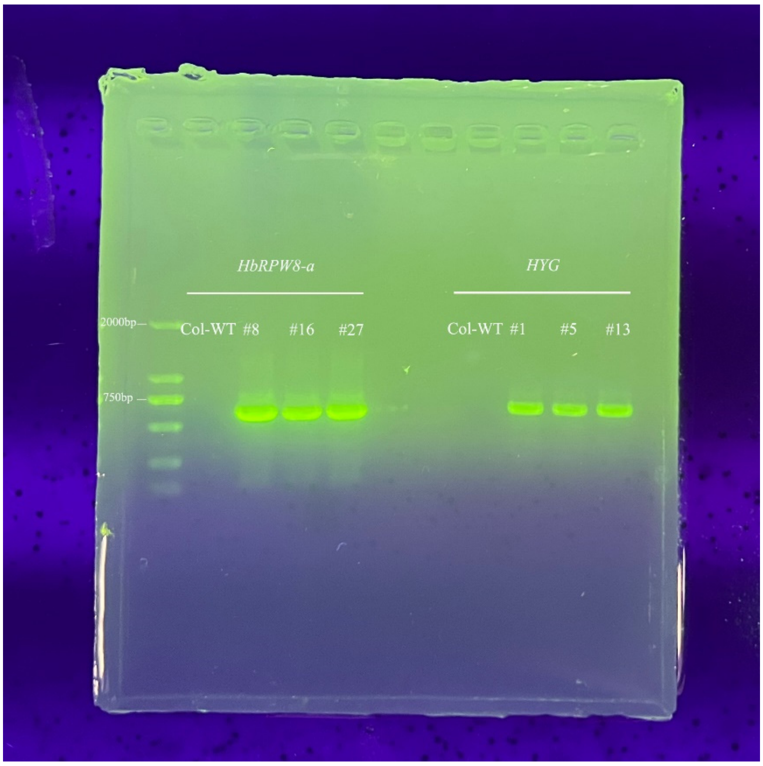

HbRPW8-a
HYG
2000bp
750bp
Col-WT #8 #16 #27
Col-WT #1 #5 #13
